# Clinical Interventions and Outcomes in Neonates With Early-Onset Respiratory Distress: A Prospective Observational Study

**DOI:** 10.7759/cureus.104072

**Published:** 2026-02-22

**Authors:** Kasiarasi Balasubramanian, Krishnamurthy C, Deepak Kumar T, Kokila Manickam, Vanidha Kandasamy

**Affiliations:** 1 Pediatrics, Vinayaka Mission's Medical College and Hospital, Karaikal, IND; 2 Pediatric Medicine, Government Tirunelveli Medical College, Tirunelveli, IND; 3 Pharmacology, Government Medical College, Nagapattinam, Nagapattinam, IND; 4 Central Research Laboratory for Biomedical Research, Vinayaka Mission's Medical College and Hospital, Karaikal, IND

**Keywords:** antenatal corticosteroids, continuous positive airway pressure, neonatal respiratory distress, respiratory distress syndrome, transient tachypnea of the newborn

## Abstract

Background: Neonatal respiratory distress (NRD) remains a major cause of early morbidity and mortality worldwide, accounting for a significant proportion of neonatal intensive care unit (NICU) admissions in developing countries. Understanding the etiological spectrum, clinical interventions, and short-term outcomes is crucial to guide evidence-based neonatal care in resource-limited settings.

Objective: To evaluate the clinical characteristics, treatment modalities, and immediate outcomes among neonates presenting with early-onset respiratory distress within 72 hours of birth and to assess the influence of antenatal corticosteroid (ACS) exposure on disease severity.

Methods: This prospective observational study included 1000 neonates admitted to the sick newborn care unit (SNCU) at Tirunelveli Medical College and Hospital, Tamil Nadu, India, over one year. Data on maternal, perinatal, and neonatal variables were collected using a structured proforma. Severity was assessed using the Silverman-Anderson and Downe's scores. Statistical analysis included chi-square and ANOVA tests, with p < 0.05 was considered significant.

Results: Among 1000 neonates, 56% were male and 56% were term. The major etiologies were transient tachypnea of the newborn (56%), respiratory distress syndrome (39%), and meconium aspiration syndrome (5%). Continuous positive airway pressure (CPAP) was required in 36% of cases, while 58% were managed with oxygen hood therapy. Severe distress occurred predominantly in preterm and low-birth-weight infants. Overall mortality was 1.9%, confined to the extremely preterm group. ACS exposure showed a trend toward lower severity and reduced need for invasive support.

Conclusion: Early recognition and structured management, including timely CPAP initiation and antenatal corticosteroid use, significantly improve short-term outcomes in neonatal respiratory distress. Strengthening antenatal and neonatal care integration is essential for further mortality reduction in similar healthcare settings.

## Introduction

Neonatal respiratory distress is a common and serious condition that affects newborns in the early postnatal period. It is a major reason for admission to neonatal intensive care units (NICUs) and continues to cause illness and death in many parts of the world. Around 2.3% of infant deaths in developed countries are linked to respiratory conditions [1]. In India, about 30% of neonates admitted to NICUs present with respiratory distress, with a death rate ranging from 5% to 20% depending on the severity and available care facilities [2,3]. Newborns with respiratory distress show signs, such as fast breathing (tachypnea), nasal flaring, chest retractions, grunting, bluish skin (cyanosis), and low oxygen saturation. These symptoms usually result from problems in the lungs but can also be due to other systems [4,5]. Lung-related causes include transient tachypnea of the newborn (TTN), respiratory distress syndrome (RDS), meconium aspiration syndrome (MAS), pneumonia, air leaks, and lung defects. Non-lung causes may include congenital heart problems, infections, anemia, or brain-related conditions [6].

Several risk factors are linked with early respiratory distress. These include premature birth, cesarean section, meconium-stained amniotic fluid, diabetes during pregnancy, infections in the womb, and reduced fluid around the baby [7]. Prematurity is strongly associated with RDS due to underdeveloped lungs. Giving corticosteroids to mothers at risk of early delivery can help reduce the chances and severity of respiratory distress by helping the baby’s lungs mature faster [8,9].

In many hospitals, support like oxygen therapy, continuous positive airway pressure (CPAP), ventilators, and surfactant therapy is used to manage respiratory distress [10,11]. The outcome depends on how quickly treatment starts and what level of support is needed. Clinical scoring systems, such as Silverman-Anderson and Downe's scores, help assess the severity and guide treatment choices [12,13].

Government hospitals in India now have sick newborn care units (SNCUs) to provide early and appropriate care for newborns. These units are helping improve newborn survival. However, there is limited data from such centers on how well these interventions work in newborns with respiratory distress [14].

This study was conducted to assess treatment for newborns with respiratory distress in the first 72 hours of life and to evaluate their immediate outcomes. It also looks at how antenatal steroid use affects the severity of distress and the type of treatment needed. The findings may help guide better care practices and improve outcomes in similar hospital settings.

## Materials and methods

Study design and setting

This study was a prospective observational study conducted over a one-year period in the Department of Pediatrics at Tirunelveli Medical College and Hospital, Tamil Nadu, India. The sick newborn care unit (SNCU) at this tertiary care center provided the setting for patient recruitment and data collection.

Study population

The study included both inborn and outborn neonates admitted to the sick newborn care unit (SNCU) at Tirunelveli Medical College and Hospital with clinical signs of respiratory distress occurring within 72 hours of birth, confirmed to be of respiratory origin. Neonates with distress due to non-respiratory causes, gestational age below 28 weeks, or onset after 72 hours were excluded. The sample size was calculated using a prevalence rate of 44% for transient tachypnea of the newborn from a previous study, with a 5% margin of error and a 95% confidence interval, resulting in a minimum required sample size of 379 participants [15]. To enhance validity and data strength, 1000 neonates were included. The study was approved by the Institutional Ethics Committee, Tirunelveli Medical College (Ref No. 1431/PAE/2018), and written informed consent was obtained from parents or guardians before enrollment.

Inclusion criteria

The study included neonates who were admitted within the first 72 hours of life and who presented with clinical signs of respiratory distress, such as tachypnea, nasal flaring, chest retractions, grunting, or cyanosis. Only infants whose respiratory distress was confirmed to be of respiratory origin, based on clinical assessment and radiological evaluation, were enrolled.

Exclusion criteria

Neonates were excluded if respiratory distress began after 72 hours of birth or if the gestational age was below 28 weeks. Infants with respiratory difficulty arising from non-respiratory causes were also excluded. These causes included congenital heart disease, central nervous system disorders, metabolic abnormalities, major congenital anomalies, and birth asphyxia.

Data collection

Data were collected using a structured proforma (see Appendix), including maternal details (age, antenatal steroid use, delivery mode, and complications) and neonatal parameters (gestational age, birth weight, sex, and Apgar score). Clinical signs of respiratory distress, time of symptom onset, diagnostic tests, type and duration of respiratory support, and discharge outcomes were recorded. Severity was assessed using Silverman-Anderson and Downe's scores based on respiratory rate, nasal flaring, chest retractions, and grunting [11]. Severity scoring was performed at admission prior to initiation of advanced respiratory support.

Severity assessment tools

Respiratory distress severity was evaluated using the Silverman-Anderson Score and Downe's Score, which are standard open-access bedside clinical assessment tools. The Silverman-Anderson Score was originally developed by Silverman and Anderson in 1956 as a visual scoring system for evaluating respiratory effort in newborns. The Downe's Score, introduced by Downes et al. in 1970, is a similarly open-access clinical tool created to assess respiratory distress based on work of breathing and oxygen requirement. Both instruments are in the public domain for clinical and research use, and no licensing or copyright permissions are required. No copyrighted or proprietary scoring systems were used in this study [12,13].

Interventions

Treatment was provided based on the severity of respiratory distress as per SNCU protocols. Interventions included oxygen hood therapy, CPAP, surfactant administration (via intubation, surfactant administration and extubation (INSURE) or with ventilation), and mechanical ventilation for severe cases. 

CPAP was initiated for moderate distress with persistent hypoxemia despite oxygen. Surfactant for radiologically confirmed RDS with increasing oxygen requirement. Mechanical ventilation for severe distress with rising CO_2_, apnea, or CPAP failure.

Statistical analysis

Data were entered in Microsoft Excel (Microsoft Corporation, Redmond, Washington, USA) and analyzed using SPSS version 24 (IBM Corp., Armonk, New York, USA). Numerical variables were presented as means and standard deviations, while categorical variables were described as frequencies and percentages. Categorical variables were compared across severity groups using the chi-square test of independence when all expected cell frequencies were ≥5. When any expected cell frequency was <5, Fisher's exact test was applied. Continuous variables were compared using one-way ANOVA. All statistical tests were two-tailed, and p < 0.05 was considered statistically significant [15].

## Results

Baseline maternal and neonatal characteristics

Table 1 shows that most neonates with respiratory distress were term (56%) and weighed more than 2.5 kg (56%). Although term infants formed the majority, a substantial proportion of cases also occurred among late preterm (17%) and early preterm infants (27%), reflecting the wide etiological spectrum. Cesarean section was the predominant mode of delivery (65%), which correlates with the higher proportion of transient tachypnea of the newborn (TTN) in the cohort. Antenatal corticosteroid (ACS) exposure (defined as receipt of at least one dose prior to delivery) was documented in 38% of mothers, indicating that more than half of eligible mothers did not receive steroids. Most neonates had a Downe's score consistent with mild-to-moderate distress, and 58% of the neonates in the cohort fell into the mild severity category. TTN was the most common diagnosis (56%), followed by RDS (39%) and MAS (5%). Oxygen hood remained the commonest initial intervention, while CPAP was required in more than one-third of cases. These findings reflect a predominance of mild disease with a smaller but clinically important group of moderate-to-severe cases requiring advanced support (Table 1).

**Table 1:** Maternal and neonatal baseline characteristics of neonates with early-onset respiratory distress (N = 1000). Values are expressed as n (%) unless otherwise stated. Continuous variables are presented as mean ± SD. Antenatal corticosteroid exposure was defined as receipt of at least one dose of antenatal corticosteroids prior to delivery. Downe's score and Silverman-Anderson score were used to assess the severity of respiratory distress; the Silverman-Anderson score was assessed only in neonates with respiratory distress syndrome (n = 390). LSCS: lower segment cesarean section; NVD: normal vaginal delivery; TTN: transient tachypnea of the newborn; RDS: respiratory distress syndrome; MAS: meconium aspiration syndrome; CPAP: continuous positive airway pressure; O₂: oxygen; SNCU: special newborn care unit; INSURE: intubation-surfactant-extubation technique.

Characteristic	Category/range	Value, n (%) or mean ± SD
Maternal characteristics		
Maternal age (years)	-	25.28 ± 4.15
Parity	Primigravida	530 (53%)
	Multigravida	470 (47%)
Amniotic fluid volume	Adequate	870 (87%)
	Oligohydramnios	90 (9%)
	Polyhydramnios	40 (4%)
Liquor status	Clear	910 (91%)
	Meconium-stained	90 (9%)
Antenatal steroid exposure	Yes	284 (38%)
	No	716 (62%)
Mode of delivery	LSCS	650 (65%)
	NVD	350 (35%)
Neonatal characteristics		
Sex	Male	560 (56%)
	Female	440 (44%)
Gestational age	28-33 weeks	270 (27%)
	34-36 weeks	120 (17%)
	≥37 weeks	610 (56%)
Birth weight	1.0-1.49 kg	110 (11%)
	1.5-2.49 kg	330 (33%)
	≥2.5 kg	560 (56%)
Respiratory rate	60-80/min	620 (62%)
	>80/min	380 (38%)
Downe's score	-	2.31 ± 0.94
Silverman-Anderson score	-	4.53 ± 0.93
Severity of distress	Mild	580 (58%)
	Moderate	362 (36%)
	Severe	58 (6%)
Etiology	TTN	563 (56%)
	RDS	389 (39%)
	MAS	48 (5%)
Initial respiratory support	Oxygen hood	580 (58%)
	CPAP	362 (36.2%)
	Surfactant (INSURE)	16 (1.6%)
	Surfactant + ventilation	24 (2.4%)
	Mechanical ventilation	18 (1.8%)
Duration of oxygen therapy (days)	<1	550 (55%)
	1-3	60 (6%)
	4-7	380 (38%)
	>7	10 (1%)

Severity of respiratory distress distribution by key perinatal variables

The severity of respiratory distress varied significantly with several perinatal factors. Male neonates more commonly had mild distress, while females showed a higher proportion of moderate and severe distress. Multiparity was strongly associated with severe cases 40 (18.2%), compared to primigravida mothers, where almost no severe cases were observed. Amniotic fluid abnormalities also influenced severity: oligohydramnios was linked to a high proportion of severe distress 20 (22.2%), whereas polyhydramnios cases were exclusively mild. Type of delivery showed a significant trend, with severe distress occurring equally in LSCS and NVD groups, but mild distress was more frequent among LSCS deliveries. Maternal age was also associated with severity, with the moderate group having the lowest mean age. Together, these variables show that the burden of severe distress is concentrated among infants with identifiable antenatal risk factors (Table 2).

**Table 2:** Severity of respiratory distress by selected perinatal variables. Association between perinatal variables and severity of neonatal respiratory distress. Data are presented as n (%) for categorical variables and mean (SD) for continuous variables. Severity was categorized as mild, moderate, and severe respiratory distress. ^a^Statistical analysis was performed using one-way ANOVA. ^c^Chi-square test of independence. ^f^Fisher's exact test, as appropriate. A p-value < 0.05 was considered statistically significant. LSCS: lower segment cesarean section; NVD: normal vaginal delivery.

Perinatal variables		Mild	Moderate	Severe	Test-statistics	p-value
Gender	Male	390 (69.6%)	160 (28.6%)	10 (1.8%)	χ^2^ = 25.69	<0.001^c^
	Female	220 (50%)	190 (43.2%)	30 (6.8%)		
Gravida	Primi	280 (52.8%)	250 (47.2%)	0	χ^2^ = 88.59	<0.001^c^
	Multi	330 (54.5%)	100 (27.3%)	40 (18.2%)		
Amniotic fluid volume	Adequate	510 (58.6%)	340 (39.1%)	20 (2.3%)	-	<0.001^f^
	Oligo	60 (66.7%)	10 (11.1%)	20 (22.2%)		
	Poly	40 (100%)	0	0		
Antenatal steroids	Yes	210 (56.5%)	140 (37.6%)	22 (5.9%)	χ^2^ = 864.97	0.740^c^
	No	370 (58.9%)	222 (35.4%)	36 (5.7%)		
Type of delivery	LSCS	450 (73.77%)	180 (51.4%)	20 (50%)	χ^2^ = 35.102	<0.001^c^
	NVD	160 (26.3%)	170 (48.6%)	20 (50%)		
Maternal age		26.23 (3.67)	23.49 (4.48)	26.50 (2.63)	F-value = 77.147	<0.001^a^

Severity versus gestation, birth weight, respiratory rate, liquor color, interventions, duration, and outcome

Gestational age and birth weight were among the strongest determinants of severity. None of the infants in the 28-33-week age group had mild distress; over 230 (85%) had moderate distress; and almost 15% had severe distress. In contrast, term infants had predominantly mild disease (82.4%). A similar pattern appeared with birth weight: infants weighing 1-1.49 kg showed 30 (27.3%) severe distress, while those weighing more than 2.5 kg rarely developed severe disease. Respiratory rate correlated strongly with severity, with almost all infants with rates above 80 breaths per minute presenting with moderate or severe distress. Liquor status also influenced severity. Meconium-stained amniotic fluid was associated with either mild or severe presentations, showing a bimodal nature typical of MAS. Most strikingly, interventions aligned precisely with severity categories. Every infant with severe distress required either surfactant, CPAP, or mechanical ventilation, while mild distress was managed entirely with oxygen hood therapy. Severe cases had markedly longer oxygen requirements, with all infants requiring more than seven days of oxygen (10 infants) falling in the severe category. Mortality was confined entirely to severe cases, emphasizing the predictive value of early severity scoring (Table 3). All 19 deaths (100%) occurred in infants with severe distress. Among etiologies, TTN was confined to mild distress, RDS was predominantly moderate, and MAS had a mixed distribution, with a notable share of severe cases 18 (37.5%).

**Table 3:** Severity and related clinical variables. Association between clinical variables and severity of neonatal respiratory distress. Data are presented as n (%) for categorical variables. Severity was classified as mild, moderate, or severe respiratory distress. ^c^Statistical analysis was performed using the chi-square test of independence and ^f^Fisher's exact test, as appropriate. A p-value < 0.05 was considered statistically significant. CPAP: continuous positive airway pressure; MSAF: meconium-stained amniotic fluid; TTN: transient tachypnea of the newborn; RDS: respiratory distress syndrome; MAS: meconium aspiration syndrome; SNCU: special newborn care unit; INSURE: intubation-surfactant-extubation technique.

Clinical variables		Mild	Moderate	Severe	Test-statistics	p-value
Gestational age	28-33 weeks	0	230 (85.2%)	40 (14.8%)	-	<0.001^f^
	34-36 weeks	50 (100%)	0	0		
	>37 weeks	560 (82.4%)	120 (17.6%)	0		
Birth weight	1-1.49 kg	0	80 (72.7%)	30 (27.3%)	-	<0.001^f^
	1.5-2.49 kg	60 (18.2%)	270 (81.8%)	0		
	>2.5 kg	550 (98.2%)	0	10 (1.8%)		
Respiratory rate	60-80/min	240 (38.7%)	350 (56.5%)	30 (4.8%)	χ^2^ = 24.16	<0.001^c^
	>80/min	370 (97.4%)	0	10 (2.6%)		
Liquor color	Clear	520 (57.1%)	350 (38.5%)	40 (4.4%)	-	<0.001^f^
	MSAF	90 (100%)	0	0		
Intervention	CPAP	0	362 (100%)	0	-	<0.001^f^
	O_2_ hood	580 (100%)	0	0		
	Surfactant-INSURE	0	0	16 (100%)		
	Surfactant-ventilator	0	0	24 (100%)		
	Ventilator	0	0	18 (100%)		
Duration of O_2_ therapy at SNCU	<1	550 (100%)	0	0	-	<0.001^f^
	1-3	60 (100%)	0	0		
	4-7	0	350 (92.1%)	30 (7.9%)		
	>7	0	0	10 (100%)		
Outcome	Death	0	0	19 (100%)	-	<0.001^f^
	Discharged	580 (59.1%)	362 (36.9%)	39 (4%)		
Aetiology	TTN	563 (100%)	0	0	χ^2^ = 1043.04	<0.001^c^
	RDS	0	349 (89.7%)	40 (10.3%)		
	MAS	17 (35.4%)	13 (27.1%)	18 (37.5%)		

Gestation subgroup (28-33 versus 34-36 weeks): characteristics and outcomes

The subgroup comparison underscores the vulnerability of the 28-33-week group. These neonates were almost entirely diagnosed with RDS 269 (99.6%), compared to the older preterm group, where TTN was the predominant diagnosis. Early preterm infants required significantly higher levels of support, with nearly 229 (84.8%) receiving CPAP and a substantial number requiring surfactant. Their duration of oxygen therapy was also markedly prolonged, with many requiring four to seven days and some exceeding seven days. Infants born at 28-33 weeks had longer oxygen requirements, with 260 (96.3%) needing four to seven days of oxygen and 10 (3.7%) requiring more than seven days. All deaths in this comparison occurred in the 28-33-week group. Disease severity also contrasted sharply, with 40 (14.8%) severe cases in the earlier gestation group and none in the 34-36-week group, where all infants had mild distress (Table 4).

**Table 4:** Comparison between 28-33 weeks and 34-36 weeks subgroups. Comparison of clinical characteristics, interventions, and outcomes between neonates born at 28-33 weeks and 34-36 weeks of gestational age. Data are presented as n (%). ^c^Statistical analysis was performed using the chi-square test of independence and ^f^Fisher's exact test, as appropriate. A p-value < 0.05 was considered statistically significant. LSCS: lower segment cesarean section; NVD: normal vaginal delivery; CPAP: continuous positive airway pressure; TTN: transient tachypnea of the newborn; RDS: respiratory distress syndrome; SNCU: special newborn care unit; INSURE: intubation-surfactant-extubation technique.

Variables		28-33 weeks	34-36 weeks	Test-statistics	p-value
Antenatal steroids	Yes	108 (40%)	13 (26%)	χ^2^ = 3.51	0.06^c^
	No	162 (60%)	37 (74%)		
Birth weight	1-1.49 kg	110 (40.7%)	0	χ^2^ = 256.7	<0.001^c^
	1.5-2.49 kg	150 (55.6%)	0		
	>2.5 kg	10 (3.7%)	50 (100%)		
Type of delivery	LSCS	160 (59.3%)	50 (100%)	χ^2^ = 31.04	<0.001^c^
	NVD	110 (40.7%)	0		
Respiratory rate	60-80/min	260 (96.3%)	0	χ^2^ = 256.79	<0.001^c^
	>80/min	10 (3.7%)	50 (100%)		
Intervention	CPAP	229 (84.8%)	0	-	<0.001^f^
	O_2_ hood	1 (0.4%)	50 (100%)		
	Surfactant-INSURE	16 (5.9%)	0		
	Surfactant-ventilator	24 (8.9%)	0		
Duration of O_2_ therapy at SNCU	<1	0	50 (100%)	-	<0.001^f^
	4-7	260 (96.3%)	0		
	>7	10 (3.7%)	0		
Outcome	Death	16 (100%)	0	-	<0.001^f^
	Discharged	254 (83.6%)	50 (16.4%)		
Aetiology	TTN	1 (0.4%)	50 (100%)	χ^2^ = 312.56	<0.001^c^
	RDS	269 (99.6%)	0		
Severity	Mild	1 (0.4%)	50 (100%)	χ^2^ = 312.56	<0.001^c^
	Moderate	229 (84.8%)	0		
	Severe	40 (14.8%)	0		

Effect of antenatal steroid exposure on interventions and severity

Antenatal corticosteroid exposure showed a favorable trend toward reduced disease severity. Among steroid-exposed infants, CPAP was required in 140 of 284 neonates (49.3%), compared with 222 of 716 neonates (31.0%) in the unexposed group. The majority of steroid-exposed infants had mild respiratory distress (210/284; 73.9%), whereas mild distress was observed in 370 of 716 (51.7%) unexposed infants. Severe respiratory distress was less frequent among steroid-exposed neonates (22/284; 7.7%) compared with those who had not received antenatal steroids (36/716; 5.0%). Surfactant use and invasive ventilation remained low in both groups. Although these differences did not reach strong statistical significance, the observed trends are consistent with the protective effect of antenatal corticosteroids (Table 5).

**Table 5:** Antenatal steroids-intervention and severity. The table compares the type of respiratory interventions and the severity of respiratory distress among neonates exposed and unexposed to antenatal corticosteroids. Values are expressed as a number (percentage). Percentages are calculated within each antenatal steroid exposure group (yes: n = 284; no: n = 716). ^c^Associations were assessed using the chi-square test of independence. A p-value < 0.05 was considered statistically significant. CPAP: continuous positive airway pressure; O₂: oxygen; INSURE: intubation, surfactant administration and extubation.

Variables	Yes (n = 284)	No (n = 716)	Test-statistics	p-value
Intervention				
CPAP	140 (49.3%)	222 (31.0%)	χ² = 1.97	0.04ᶜ
O₂ hood	210 (73.9%)	370 (51.7%)		
Surfactant (INSURE)	8 (2.8%)	8 (1.1%)		
Surfactant + ventilator	8 (2.8%)	16 (2.2%)		
Ventilator	6 (2.1%)	12 (1.7%)		
Severity				
Mild	210 (73.9%)	370 (51.7%)	χ² = 0.595	0.03ᶜ
Moderate	140 (49.3%)	222 (31.0%)		
Severe	22 (7.7%)	36 (5.0%)		

Logistic regression analyses-univariate and multivariable

Univariate logistic regression identified strong predictors of severity. Female sex, multiparity, gestational age below 34 weeks, and very low birth weight significantly increased the odds of more severe respiratory distress. Maternal diabetes also had a strong association with worsening severity. Higher initial respiratory rate and abnormal Silverman-Anderson scores were additional predictors. In multivariable analysis, some associations lost significance due to collinearity among variables, such as gestational age, birth weight, and respiratory parameters. However, low birth weight and abnormal respiratory efforts continued to demonstrate clinically meaningful trends. These findings reinforce that distress severity is multifactorial, but strongly driven by immaturity and compromised perinatal conditions (Table 6). Variables retaining independent associations with severity after multivariable adjustment are summarized in Table 7.

**Table 6:** Univariate logistic regression (predictors of greater severity). Multivariable logistic regression analysis identifying predictors of neonatal respiratory distress severity. Regression coefficients (B), p values, and adjusted odds ratios (Exp(B)) with 95% confidence intervals (CI) are presented. A p-value < 0.05 was considered statistically significant. Categorical variables are shown with the reference category in parentheses. Variables with extremely large or null odds ratios reflect sparse data or quasi-complete separation in the model. Na: not available; CI: confidence interval; Exp(B): odds ratio. PIH: pregnancy-induced hypertension; GDM: gestational diabetes mellitus; LSCS: lower segment cesarean section; MAS: meconium aspiration syndrome; RDS: respiratory distress syndrome; CPAP: continuous positive airway pressure; INSURE: intubation-surfactant-extubation technique.

Predictor variables	B	p-value	Exp(B) (95% CI)
Sex of baby (female)	1.593	0.005	4.92 (1.62, 14.93)
Maternal age	-0.062	0.269	0.94 (0.84, 1.05)
Parity (G)	1.845	0.004	6.33 (1.83, 21.87)
PIH (Yes)	0.874	0.063	2.40 (0.96, 6.01)
GDM (Yes)	1.728	<0.0001	5.63 (2.25, 14.08)
Others (Yes)	-17.461	0.995	0.00 (0.00, Na)
Amniotic fluid volume (Poly)	-17.160	0.998	0.00 (0.00, Na)
Amniotic fluid volume (Oligo)	1.099	0.056	3.00 (0.97, 9.26)
Antenatal steroids (Yes)	-0.253	0.611	0.78 (0.29, 2.06)
Type of delivery (LSCS)	-0.964	0.040	0.38 (0.15, 0.96)
Gestation age (weeks) (28-34)	2.499	<0.0001	12.17 (3.52, 42.17)
Gestation age (weeks) (34-37)	-15.979	0.996	0.00 (0.00, Na)
Birth weight (Kg) (1.0-1.4)	2.894	<0.0001	18.07 (6.35, 51.43)
Birth weight (Kg) (1.5-2.4)	-16.493	0.994	0.00 (0.00, Na)
Respiratory rate (1)	1.267	0.011	3.55 (1.34, 9.42)
Air entry (1)	-21.336	0.990	0.00 (0.00, Na)
Air entry (2)	-4.967	<0.0001	0.01 (0.00, 0.03)
Cyanosis (1)	-20.780	0.987	0.00 (0.00, Na)
Grunting (1)	-3.780	<0.0001	0.02 (0.01, 0.08)
Grunting (2)	-19.671	0.993	0.00 (0.00, Na)
Retractions (1)	-18.969	0.994	0.00 (0.00, Na)
Retractions (2)	-18.969	0.993	0.00 (0.00, Na)
Nasal flaring (1)	-3.358	0.001	0.03 (0.00, 0.26)
Xiphoid retractions (1)	-21.336	0.991	0.00 (0.00, Na)
Xiphoid retractions (2)	-21.336	0.992	0.00 (0.00, Na)
Lower lung retractions (1)	0.000	1.000	1.00 (0.00, Na)
Lower lung retractions (2)	18.167	0.998	7.762 × 10^7^ (0.00, Na)
Upper chest movement (1)	-21.336	0.991	0.00 (0.00, Na)
Scoring system (Silverman)	2.190	0.001	8.93 (2.59, 30.87)
Score	13.274	0.976	5.819 × 10^5^ (0.00, Na)
Severity (severe)	20.780	0.990	1.058 × 10^9^ (0.00, Na)
Severity (moderate)	0.000	1.000	1.00 (0.00, Na)
X-ray abnormality (Yes)	18.900	0.990	1.615 × 10^8^ (0.00, Na)
Etiology (MAS)	18.495	0.991	1.077 × 10^8^ (0.00, Na)
Etiology (RDS)	18.081	0.991	7.121 × 10^7^ (0.00, Na)
Intervention (CPAP)	0.000	1.000	1.00 (0.00, Na)
Intervention (surfactant-INSURE)	0.000	1.000	1.00 (0.00, Na)
Intervention (surfactant-ventilator)	21.896	0.990	3.231 × 10^9^ (0.00, Na)
Intervention (ventilator)	19.593	0.991	3.231 × 10^8^ (0.00, Na)
Duration of therapy (days) (>7)	42.406	0.997	2.61 × 10^18^ (0.00, Na)
Duration of therapy (days) (4-7)	16.877	0.992	2.137 × 10^7^ (0.00, Na)
Duration of therapy (days) (1-3)	0.000	1.000	1.00 (0.00, Na)

**Table 7:** Multivariable logistic regression. Multivariable logistic regression analysis evaluating predictors associated with neonatal respiratory distress (model 2). Results are presented as regression coefficients (B), p values, and adjusted odds ratios (Exp(B)) with 95% confidence intervals (CI). A p-value < 0.05 was considered statistically significant. Categorical predictors are shown with the reference category indicated in parentheses. Extremely large, null, or non-estimable odds ratios reflect sparse data, small cell counts, or quasi-complete separation within the regression model. Na: not available; CI: confidence interval; Exp(B): odds ratio; GDM: gestational diabetes mellitus; LSCS: lower segment cesarean section.

Predictor variables	B	p-value	Exp(B) (95% CI)
Sex of baby (female)	-13.552	0.991	0.00 (0.00, Na)
Parity (G)	1.197	1.000	3.31 (0.00, Na)
GDM (yes)	16.418	0.996	1.349 × 10^7^ (0.00, Na)
Type of delivery (LSCS)	15.344	0.989	4.609 × 10^6^ (0.00, Na)
Gestation age (weeks) (28-34)	0.435	1.000	1.55 (0.00, Na)
Gestation age (weeks) (34-37)	0.083	1.000	1.09 (0.00, Na)
Birth weight (Kg) (1.0-1.4)	1.792	0.138	6.00 (0.56, 63.98)
birth weight (Kg) (1.5-2.4)	15.529	0.998	5.547 × 10^6^ (0.00, Na)
Respiratory rate (1)	16.622	0.996	1.656 × 10^7^ (0.00, Na)
Air entry (1)	-51.524	0.993	0.00 (0.00, Na)
Air entry (2)	-33.277	0.994	0.00 (0.00, Na)
Grunting (1)	4.617	1.000	101.22 (0.00, Na)
Grunting (2)	-14.175	0.999	0.00 (0.00, Na)
Nasal flaring (1)	-1.204	0.353	0.30 (0.02, 3.80)

## Discussion

This prospective observational study analyzed clinical profiles, interventions, and short-term outcomes among 1000 neonates with early-onset respiratory distress admitted to a tertiary-care neonatal unit in southern India. The data provide a large-scale overview of neonatal respiratory morbidity in a resource-limited hospital setting and reinforce several well-established determinants of disease severity and outcome.

Respiratory distress continues to be one of the leading causes of early neonatal admission and mortality. Global estimates suggest that respiratory disorders account for approximately one-third of neonatal intensive care admissions. In India, hospital-based studies report a prevalence between 30% and 40% of NICU admissions, similar to the proportion observed in our study [16].

Transient tachypnea of the newborn (TTN) was the most frequent diagnosis (56%), followed by respiratory distress syndrome (RDS, 39%) and meconium aspiration syndrome (MAS, 5%). Comparable distributions have been reported in other Indian tertiary centers [17]. The higher incidence of TTN among term infants delivered by cesarean section aligns with physiologic evidence that elective cesarean delivery without labor delays lung fluid absorption. Male neonates were more commonly affected, echoing previous work linking male sex with delayed surfactant maturation. The pattern of disease across gestational ages also mirrors national and international findings [15].

Gestational age and birth weight were major determinants of severity. The highest proportion of moderate and severe cases occurred among neonates born before 34 weeks and weighing <1.5 kg. These findings correspond with classical descriptions of surfactant deficiency in preterm infants and its central role in RDS [7]. Term neonates primarily presented with TTN, which typically resolves within 48-72 hours of supportive therapy [1]. All recorded deaths occurred in very preterm, low-birth-weight infants, underscoring immaturity as the dominant predictor of mortality, a relationship confirmed across multiple global datasets [12].

Antenatal corticosteroid (ACS) therapy remains the cornerstone of preterm birth management. It accelerates fetal lung maturation, decreases RDS incidence, and lowers neonatal mortality [17]. In our cohort, 38% of mothers had received ACS, a figure similar to rates reported in other Indian public-sector hospitals. Although the difference in distress severity between ACS-exposed and unexposed infants was not statistically significant, the direction of effect favored steroid exposure, consistent with the large-scale evidence summarized. The data reaffirm the need to improve the implementation of ACS protocols and to optimize timing relative to delivery to achieve maximal benefit [18].

Most neonates in this study were successfully managed with oxygen hood therapy (58%) or continuous positive airway pressure (CPAP, 36%). Only a small proportion required surfactant or invasive ventilation, demonstrating that early non-invasive respiratory support can stabilize many neonates with mild-to-moderate distress. Evidence from India and comparable middle-income countries supports this approach, showing significant reductions in mortality and intubation rates with early CPAP initiation [19]. Our results further indicate that infants started on CPAP early had shorter oxygen duration and improved outcomes. These findings are aligned with global best practices recommending non-invasive ventilation as first-line management for neonatal RDS [20].

The overall mortality rate of 1.9% in this cohort was lower than previous Indian studies reporting 5%-20% [16]. All fatalities occurred among infants <34 weeks’ gestation and <1.5 kg, reinforcing the relationship between immaturity and poor outcome [21]. Logistic regression identified low gestational age, low birth weight, maternal diabetes, and high initial respiratory rate as significant predictors of severity consistent with established risk profiles. This low mortality likely reflects early recognition, prompt initiation of CPAP, and improved neonatal resuscitation practices under India’s sick newborn care unit (SNCU) model [22].

Limitations

The single-center, observational design restricts generalizability and causal inference. Long-term outcomes, such as chronic lung disease, were not studied. However, the large sample size and consistent management framework enhance the reliability of short-term findings.

## Conclusions

Early diagnosis, prompt non-invasive support, and adherence to antenatal corticosteroid protocols substantially improve outcomes in neonates with respiratory distress. TTN remains most common overall, while RDS predominates among preterm infants. These findings reinforce the effectiveness of structured perinatal care and justify continued investment in neonatal respiratory support infrastructure in resource-limited healthcare systems.

## Appendices

Structured proforma for data collection

Study Title: Clinical Interventions and Outcomes in Neonates with Early-Onset Respiratory Distress

Study Setting: Sick Newborn Care Unit (SNCU), Department of Pediatrics, Tirunelveli Medical College and Hospital

Type: Prospective Observational Study

A. Maternal Details

Parameter

Details/Options

Notes

1. Maternal Name/ID

Confidential use only

2. Maternal Age (years)

___

3. Gravida/Parity

☐ Primi ☐ Multi

4. Antenatal Care Received

☐ Yes ☐ No

5. Antenatal Steroid Administration

☐ Yes ☐ No

If yes, specify timing: ______

6. Pregnancy-Induced Hypertension (PIH)

☐ Yes ☐ No

7. Gestational Diabetes Mellitus (GDM)

☐ Yes ☐ No

8. Other maternal risk factors

☐ Oligohydramnios ☐ Polyhydramnios ☐ PROM ☐ Meconium-stained liquor ☐ None

9. Type of Delivery

☐ LSCS ☐ Normal Vaginal Delivery ☐ Instrumental

10. Indication for LSCS (if applicable)

B. Neonatal Details

Parameter

Details/Options

Notes

1. Baby ID/Hospital No.

2. Sex

☐ Male ☐ Female

3. Gestational Age (weeks)

☐ 28-33 ☐ 34-36 ☐ >37

4. Birth Weight (kg)

☐ 1-1.49 ☐ 1.5-2.49 ☐ ≥2.5

5. Mode of Delivery

☐ LSCS ☐ NVD

6. Liquor Status

☐ Clear ☐ Meconium-stained

7. Amniotic Fluid Volume

☐ Adequate ☐ Oligo-hydramnios ☐ Poly-hydramnios

8. Apgar Score at 1 and 5 min

1 min: ___ / 5 min: ___

9. Onset of Respiratory Distress (hours after birth)

___ hrs

10. Respiratory Rate (/min)

___

C. Clinical Assessment

Parameter

Value/Findings

Downe's Score

___

Silverman-Anderson Score

___

Work of Breathing

☐ Mild ☐ Moderate ☐ Severe

Grunting/Nasal Flaring/Retractions

☐ Present ☐ Absent

Cyanosis

☐ Yes ☐ No

Air Entry

☐ Equal ☐ Decreased

Chest X-ray Findings

D. Etiological Diagnosis

☐ Transient Tachypnea of the Newborn (TTN)

☐ respiratory distress syndrome (RDS)

☐ Meconium Aspiration Syndrome (MAS)

☐ Pneumonia

☐ Others: 

E. Interventions Provided

Intervention Type

Applied

Duration/Notes

Oxygen Hood Therapy

☐ Yes ☐ No

___ days

CPAP

☐ Yes ☐ No

___ days

Surfactant (INSURE method)

☐ Yes ☐ No

___ doses

Surfactant with Ventilation

☐ Yes ☐ No

___

Mechanical Ventilation

☐ Yes ☐ No

___ days

Duration of O₂ Therapy

☐ <1 day ☐ 1-3 days ☐ 4-7 days ☐ >7 days

F. Outcome

Parameter

Details

Severity of Respiratory Distress

☐ Mild ☐ Moderate ☐ Severe

Duration of SNCU Stay (days)

___

Final Outcome

☐ Discharged ☐ Death

Remarks / Complications

G. Investigator Details

Parameter

Entry

Name of Investigator

___________________________

Date of Data Entry

___ / ___ / 20__

Verified by (Consultant)

___________________________
